# EGF Stimulates Rab35 Activation and Gastric Cancer Cell Migration by Regulating DENND1A-Grb2 Complex Formation

**DOI:** 10.3389/fphar.2018.01343

**Published:** 2018-11-22

**Authors:** Bixing Ye, Biao Duan, Wenjie Deng, Yueyuan Wang, Yan Chen, Jie Cui, Shixiu Sun, Yujie Zhang, Jun Du, Luo Gu, Lin Lin, Yurong Tang

**Affiliations:** ^1^Department of Gastroenterology, The First Affiliated Hospital of Nanjing Medical University, Nanjing, China; ^2^Department of Physiology, Nanjing Medical University, Nanjing, China; ^3^Department of Biochemistry and Molecular Biology, Nanjing Medical University, Nanjing, China; ^4^Department of Physiology, Xuzhou Medical University, Xuzhou, China; ^5^Jiangsu Key Lab of Cancer Biomarkers, Prevention and Treatment, Collaborative Innovation Center for Cancer Personalized Medicine, Nanjing Medical University, Nanjing, China

**Keywords:** Rab35, DENND1A, Grb2, interaction, cell migration, gastric cancer, prognosis

## Abstract

**Aims:** The aim of this study was to reveal the specific molecular mechanisms by which DENND1A accepts EGF signaling and activates Rab35 in gastric cancer.

**Methods:** The expression of proteins related to DENND1A was examined by western blot analysis. Activation of Rab35 was assessed by GST-pulldown. The interaction of DENND1A and Grb2 was assessed by GST-pulldown and co-immunoprecipitation assays. The relationship between DENND1A and cell migration and invasion was detected using wound healing and transwell by gene overexpression and RNA interference.

**Results:** EGF stimulation significantly promoted cell migration, whereas transfection with siRab35 partially inhibited EGF-promoted cell migration. DENND1A is also involved in these processes and active Rab35. Moreover, DENND1A binds to the N-terminal and C-terminal SH3 domains of Grb2 through PRD. Of special interest is the observation that EGFR can recruit Grb2-DENND1A complex under EGF stimulation. Further results reveal that the higher the expression of DENND1A, the shorter progression-free survival of gastric cancer patients.

**Conclusion:** In summary, we confirmed that EGF-Grb2-DENND1A-Rab35 signaling pathway with the interaction of DENND1A and Grb2 as a regulatory center could regulate gastric cancer cell migration and invasion. Ultimately, the expression level of DENND1A predicts the survival status of gastric cancer patients and may become one of the important targets for the treatment of gastric cancer.

## Introduction

Gastric cancer is one of the most common malignancies. According to statistics, in 2012, there were 952,000 new cases of gastric cancer in the world, and 723,000 people died which accounted for the 4th and 2nd in the incidence and death of cancer (Ferlay et al., 2015). China is the country with the highest burden of gastric cancer. New cases and deaths of gastric cancer in China account for 44% (42.0 million) and 41% (297,000) of the world’s cancers, respectively, accounting for the third most common cause of cancer and death in China (Chen et al., 2015). This shows that gastric cancer is one of the major diseases that seriously threatens the lives and health of people. The metastasis of gastric cancer is the main cause of death of patients. Therefore, it is of great significance to study the mechanism of gastric cancer metastasis in the prevention and treatment of gastric cancer.

Epidermal growth factor receptor (EGFR) is one of the tyrosine kinase receptors (RTKs) and plays an important role in the occurrence and development of gastric cancer (Birkman et al., 2016). EGFR is highly expressed in gastric cancer tissues, and gastric cancer patients overexpressing EGFR are more likely to have distant metastases, and the tumors have a higher recurrence rate and a shorter survival period (Cardoso et al., 2014; Hong et al., 2014; Jacome et al., 2014). Similarly, *in vitro* experiments, EGFR can induce proliferation and migration of gastric cancer cells, and silent expression of EGFR can significantly inhibit the proliferation and migration of gastric cancer cells (Yuan et al., 2017). Under its ligand EGF stimulation, EGFR triggers a series of downstream signaling pathways, including PI3K-AKT, Ras-MAPK, and signal transducers and activators of transcription (STAT) signaling pathways, which are involved in many stages of tumor progression, such as cell proliferation, angiogenesis, invasion, migration, metastasis and apoptosis (Hong et al., 2014). Recently, a report on Science published that shRNA screens for molecules that affect the AKT phosphorylation. It was found that Rab35 can serve as downstream of growth factor receptors including EGFR and upstream of phosphoinositide-dependent protein kinase-1 (PDK1) and mTORC2 promotes AKT phosphorylation by binding to PI3K. More importantly, Rab35 somatic mutations (Rab35^A146T^ and Rab35^F156L^) are found in various tumor cells of the uterus, lymph and lungs. And, these mutations and continuously activated Rab35^Q67L^ mutation can inhibit apoptosis, suggesting that Rab35 is involved in EGFR-mediated tumor progression (Wheeler et al., 2015).

Rab35 is a member of the Ras superfamily of small G proteins and is widely expressed in tissues. It has a variety of biological functions such as cell migration, axonal growth, cell division, and endosomal transport and circulation (Klinkert and Echard, 2016). In addition to the report of the role of Rab35 in the promotion of cancer by Wheeler DB et al., other studies have also reported that Rab35 can promote tumor metastasis. In lymphoma cells, Rab35 interacts with MPM-ALK to promote tumor progression (Crockett et al., 2004). In the cell lines, it was found that Rab35 is overexpressed and inhibits the p53 kinase PRPK, thereby regulating the cell cycle progression of tumor cells (Abe et al., 2006). In breast cancer cells, Rab35 was found to act as downstream of Wnt5a and DVL2, upstream of Rac1, and promoted cell migration (Zhu et al., 2013). Similarly, another study found that EGF stimulated Rab35 activation can promote cells migration through its effector molecule Mical-1 (Deng et al., 2016). In lung cancer cells, Rab35 was found to act as downstream of EGF to promote the formation of RUSC2-GIT2 complex, leading to directed cell migration (Duan et al., 2016). However, contrary to the above results, part of the literature found that Rab35 inhibits tumor progression. In colon cancer cells, ACAP2, a downstream effector of Rab35, and also GTPase activating proteins (GAP) of ARF6, inhibits ARF6 and thus inhibits epithelial-mesenchymal transition (EMT) of tumor cells (Allaire et al., 2013). In cervical cancer cells, miR-720 negatively regulates Rab35 and promotes cell migration (Tang et al., 2015). Thus, Rab35 has a more complex role in tumors and may be related to cell types and different stimuli in the outside resulting in different signaling pathways. Currently, Rab35 has not been reported in gastric cancer metastasis.

As a molecular switch, Rab35 has two molecular structures, Rab35-GTP activated (molecular open) and Rab35-GDP inactive (molecular off). Guanine nucleotide exchange factor (GEF) is a key molecule that promotes the activation of small G proteins and can catalyze GDP exchange as GTP (Bos et al., 2007). Currently, there are four known Rab35 GEFs: connecdenn1 (DENND1A), connecdenn2 (DENND1B), connecdenn3 (DENND1C), and folliculin (FLCN). They all contain highly conserved differentially expressed in normal and neoplastic cells domains (DENN), which is the activated Rab35 catalytic domain (Marat and McPherson, 2010; Chaineau et al., 2013). Under normal circumstances, GEF generates steric hindrance through its own intramolecular interactions, which hinders the catalytic action of the DENN domain. This phenomenon is called “self-inhibition.” The “self-inhibitory” state is released by autophosphorylation, binding to proteins or liposomes, and binding to second messengers (Cherfils and Zeghouf, 2013). In gastric cancer cells, the mechanism by which GEF participates in Rab35 regulation is not clear.

Therefore, the main purpose of this study is to observe the changes of Rab35 activity and its relationship with gastric cancer cell migration stimulated by EGF and to determine what kind of GEF is involved in the changes of Rab35 activity in gastric cancer cells and its possible mechanism.

## Materials and Methods

### Cell and Cell Culture

The human embryonic kidney cell HEK293T, normal gastric epithelial cell line GES-1 and human gastric cancer cells BGC-823 and SGC-7901 were obtained from the Cell Biology Institute of Chinese Academy of Sciences (Shanghai, China). The cells were cultured in Dulbecco’s modified Eagle’s medium containing 10% (v/v) fetal bovine serum (FBS) (Gibco, Thermo Fisher Scientific, Grand Island, NY, United States) in a humidified incubator at 37°C with 5% CO_2_. Cells were grown on plastic dishes for subsequent experiments.

### Plasmids and siRNAs

Full-length and cDNA fragments of DENND1A and Grb2 were amplified from cDNA. The polymerase chain reaction (PCR) products were cloned into the pCMV-N-HA and pCMV-N-Flag (Beyotime, Nantong, China). pEGFP-Rab35 (WT and Q67L) plasmids were kindly provided by Dr. Matthew P. Scott (Department of Medicine, Stanford University, Stanford, CA, United States). Full-length Rab35 cDNA was amplified from the Rab35 WT plasmid. The PCR product was cloned into the pGEX-4T-1. The cells were seeded in 6-well plates, cultured to 80–90% confluence, and then transiently transfected with the plasmid by ExFect^TM^ Transfection Reagent (Vazyme Biotech, Piscataway, NJ, United States) according to the transfection method provided by the manufacturer.

Duplex oligonucleotides were chemically synthesized and purified by GenePharma (Shanghai, China). The small interfering RNA (siRNA) duplexes used were DENND1A, #1, 5′-GGAGCGAAGAGCUGCUUCUdTdT-3′, #2, 5′-CCCGACCGCCUCCCAAGAUdTdT-3′ and #3, 5′-CCACCCGACCGCCUCCCAAdTdT-3′; and Rab35, #1, 5′-GCAGCAACAACAGAACGAUdTdT-3′, #2, 5′-GCUCACGAAGAACAGUAAAdTdT-3′ and #3, 5′-GAUGAUGUGUGCCGAAUAUdTdT-3′. These three kinds of siRNAs were mixed (siRNA pool) to achieve the interference effect while eliminating the off target effect through reducing the use of each kind of siRNA. Cells were transfected with siRNA duplexes using Lipofectamine 3000 (Invitrogen) according to the transfection method provided by the manufacturer.

### Reagents and Antibodies

EGF was purchased from American R&D Company (Minneapolis, MN, United States). Cycloheximide (CHX) was purchased from Sigma-Aldrich Corporation (United States). Anti-human DENND1A monoclonal antibody, anti-human Grb2 monoclonal antibody, anti-human HA monoclonal antibody and anti-EGFR monoclonal antibody were purchased from Cell Signaling Technology (Danvers, MA, United States). Normal mice IgG, normal rabbit IgG and anti-GAPDH polyclonal antibody were purchased from Santa Cruz Biotechnology (Santa Cruz, CA, United States). Anti-GST antibody was purchased from cwbiotech (Beijing, China) and anti-Rab35 polyclonal antibody was purchased from Biogot Technology (Nanjing, Jiangsu, China). Anti-Flag monoclonal antibody and FITC-labeled secondary antibody were purchased from Sigma-Aldrich Corporation. TRITC-labeled and horseradish peroxidase (HRP)-labeled secondary antibodies were purchased from Jackson ImmunoResearch (United States).

### RT-PCR

Total RNA were isolated with TRIzol reagent (Invitrogen). Equal amounts of RNA (1 μg) from each sample were used for cDNA synthesis using HiScriptQ RT SuperMix for qPCR (Vazyme, Nanjing, China). RT-PCR was performed on the ABI StepOne Real-Time PCR System (Applied Biosystems, Foster City, CA, United States) using GoTaq qPCR Master Mix assay (Promega) and analyzed using StepOne Software v2.1 (Applied Biosystems). 2^-ΔΔCt^ method was used to calculate gene expression levels. For sample loading control, GAPDH were tested.

### Western Blotting Analysis

Sample protein extraction and concentration determination of whole cells were performed as previously described (Duan et al., 2016). Briefly, equal amounts of protein were run on SDS polyacrylamide gels and transferred to nitrocellulose membrane. The resulting blots were blocked with 5% non-fat dry milk and incubated with primary antibodies overnight at 4°C. Then protein bands were detected by incubating with HRP-conjugated secondary antibodies (Santa Cruz, CA, United States) for 1–2 h at room temperature and visualized with ECL reagent (Millipore, Billerica, MA, United States) by ChemiDoc XRS+gel imaging system (Bio-Rad, United States). Densitometry analysis was performed using Quantity One software, and band intensities were normalized to those of GAPDH.

### *In vitro* Pull Down Assay and Immunoprecipitation

Cells were lysed with cell lysis buffer (20 mM Tris pH 7.5, 150 mM NaCl, 1% Triton X-100, 2.5 mM sodium pyrophosphate, 1 mM EDTA, 1% Na3VO4, 0.5 μg/ml leupeptin, 1 mM PMSF). For *in vitro* binding assays, GST fusion proteins were first purified on MagneGST glutathione particles (Promega, Madison, WI, United States). 500 μg of cell lysates transfected with GST fusion proteins was then incubated with 500–800 μg of cell lysates transfected with target proteins. For coprecipitation assays, 500 μg of cell lysates was subjected to immunoprecipitation, and proteins precipitated were detected by western blot, as described previously (Duan et al., 2016). Antibodies were used at dilutions of 1:50 or 1:100 for immunoprecipitation.

### Immunofluorescence Microscopy

Immunofluorescent was performed as previously described (Duan et al., 2016). Briefly, cells were plated on coverslips and treated with or without EGF for the time indicated after serum starvation overnight, fixed in ice-cold 4% paraformaldehyde (PFA) for 20 min, rinsed with phosphate-buffered saline (PBS) for three times and permeabilized with 0.1% Triton X-100 before blocking in 1% BSA for 1 h. The cells were incubated with primary antibodies at 4°C overnight, and then incubated with fluorescently labeled secondary antibody for 1 h at 37°C. After wash with PBS, the samples were mounted with DAPI (Southern Biotech, Birmingham, AL, United States). Then, cells were imaged by FV10i confocal fluorescence microscope (OLYMPUS, Japan).

### Transwell Assay

The transwell cell migration system consisted of cell culture champers with polycarbonate membrane inserts (8 μm pore size) in a 24-well plate. Cells were seeded on plates and transfected with siRNA for 24 h. After incubation, cells were serum starved overnight and resuspended with DMEM, and 2 × 10^4^ cells were separately seeded in the champers. Cells were allowed to attach to the membrane for 30 min. The lower chamber was filled with 500 μl DMEM with 10% FBS. After migrating for 12 h, cells that had migrated to the lower surface were fixed for 20 min and stained with 0.1% crystal violet for 15 min. The pictures were taken by Nikon TS100 (Tokyo, Japan) and counted by Image J software. All assays were performed at least three times.

### Monolayer Wound-Healing Assay

For two-dimensional cell migration assays, cells grown as described above were plated in plates on glass cover slips. When the cells reached confluence, they were incubated overnight in DMEM and pretreated with mitomycin for 1 h, and then wounding was performed by scraping through the cell monolayer with a 200 μl pipette tip. Medium and non-adherent cells were removed, and cells were washed twice with PBS and new medium with or without EGF were added for various time periods. Images were collected at the 0 h time point using an inverted microscope (Carl Zeiss Meditec, Jena, Germany). Cells were permitted to migrate into the area of clearing for 12 and 24 h in an incubator. And then, cells were removed from the incubator and imaged using the same inverted microscope. Care was taken to align the scratch along the y axis of the camera to aid subsequent image quantification.

### Immunohistochemistry

Gastric cancer tissue chips used were purchased from Shanghai Core Super Biotechnology Co., Ltd. (Shanghai, China). Ten human gastric cancer tissue and ten normal gastric tissue were used for immunohistological staining. Detailed case information can be found in Supplementary Table S1. The paraffin section were deparaffinized and rehydrated. Peroxidase blocking was done with 3% H_2_O_2_ in methanol for 15 min at 37°C. Antigen retrieval was performed by transferring the sections into Tris-acetate-ethylenediamine tetraacetic acid (EDTA) buffer (pH 8.0) inside a 700-watt microwave on full power for 5 min. After cooled down to room temperature, the sections were blocked in serum for 1 h and applied with DENND1A antibody at 4°C overnight. Then the sections were treated with the secondary antibody (1:1000) for 1 h at 37°C, and then washed in PBS. DAB substrate solution was applied to reveal the color of antibody staining. After counterstained with haematoxylin, the slides were mounted by neutral gum.

### Statistical Analysis

Data were presented as mean ± standard error of the mean (SD). Statistical analyses were performed using GraphPad Prism 5.0 software (GraphPad Software, United States). Student’s *t*-test was used for comparison between two groups, and analysis of variance was used for comparison among groups. Values of *P* < 0.05 were considered statistically significant. All experiments were repeated at least three times.

## Results

### EGF Promotes BGC-823 Migration by Activating Rab35

**FIGURE 1:**
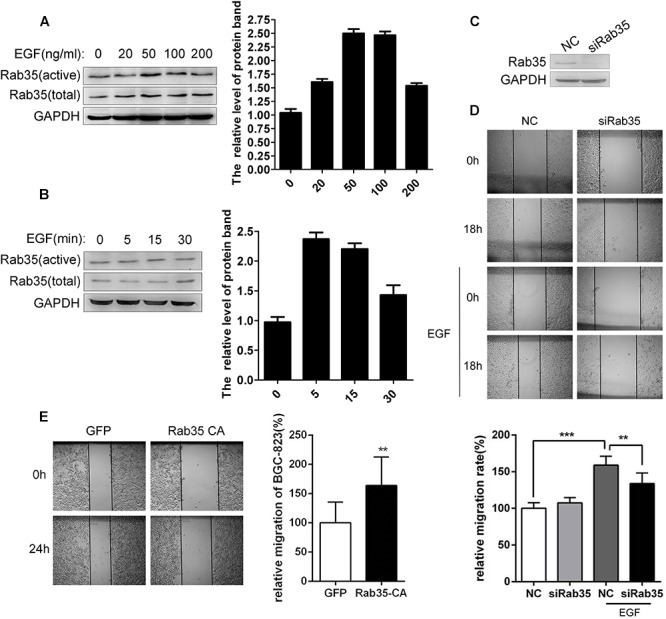
EGF promotes migration of BGC-823 by activating Rab35. **(A)** BGC-823 cells were serum-starved for 12 h and treated with EGF for the indicated concentration or **(B)** time. The endogenous activated Rab35 was precipitated by GST-RBD35. The expression of Rab35 was analyzed by western blot. **(C)** BGC-823 cells were transfected with Rab35 siRNAs pool or negative control siRNA (NC). Then, cells were lysed and analyzed for expression of Rab35 and GAPDH. **(D)** Images were taken at time 0 h and after 18 h or **(E)** 24 h of wounding. The relative migration rate was calculated by normalizing the values obtained for the NC or GFP group at 18 or 24 h as 100%. All experiments were repeated at least three times (^∗∗^*P* < 0.01; ^∗∗∗^*P* < 0.001).

Our previous studies have demonstrated that EGF can stimulate Rab35 activation in lung cancer and ovarian cancer cells (Duan et al., 2016; Zheng et al., 2017). Similarly, our study also found that EGF stimulation (50 ng/mL) for 5 min significantly increased the activity of Rab35 in BGC-823 (Figures 1A,B). Wound healing assay demonstrated that EGF stimulation significantly promoted BGC-823 cell migration, whereas transfection with siRab35 partially inhibited EGF-promoted migration (Figures 1C,D). In contrast, transfection of the Rab35-CA significantly increased BGC-823 cell migration (Figure 1E). The above results suggest that EGF promotes the migration of gastric cancer cell BGC-823 by stimulating the activation of Rab35.

### DENND1A Mediates Activation of Rab35 Upon EGF Stimulation

**FIGURE 2:**
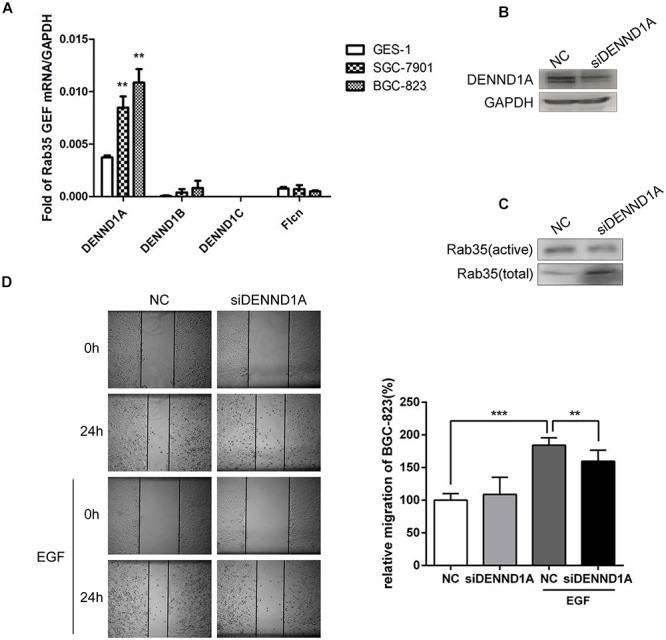
DENND1A mediates activation of Rab35 under EGF stimulation. **(A)** The expression of DENND1A, DENND1B, DENND1C, and FLCN were analyzed by RT-PCR in normal gastric epithelial cells GES-1 and gastric cancer cells BGC-823 and SGC-7901. **(B)** BGC-823 cells were transfected with DENND1A siRNAs pool or NC. Then, cells were lysed and analyzed for expression of DENND1A and GAPDH or **(C)** activated and total Rab35. **(D)** The relative migration rate of BGC-823 cells treated with or without EGF was calculated by normalizing the values obtained for the NC group without EGF at 24 h as 100%. All experiments were repeated at least three times (^∗∗^*P* < 0.01; ^∗∗∗^*P* < 0.001).

At present, there are four GEFs that can regulate the activity of Rab35. Different GEFs are specifically expressed in different tissues. However, the expression of these GEFs for Rab35 in gastric cancer tissues is not yet clear. Based on this, we detected the mRNA expression of GEFs in normal gastric epithelial cells GES-1 and gastric cancer cells BGC-823 and SGC-7901 by qPCR. The results showed that the expression of DENND1A in BGC-823 and SGC-7901 was significantly increased compared with GES-1, while the other three GEFs showed low expression in GES-1, BGC-823, and SGC-7901 with no significant difference (Figure 2A).

In view of this, we examined the effect of DENND1A on Rab35 activity in BGC-823. The results showed that siRNA interference with DENND1A significantly reduced the activity of Rab35 protein and inhibited the activation of Rab35 (Figures 2B,C). At the same time, wound healing assay demonstrated that transfection with siDENND1A significantly inhibited EGF-promoted BGC-823 cells migration (Figure 2D). These results indicate that EGF-stimulated activation of Rab35 is mediated by DENND1A.

### DENND1A and Grb2 Bind to Each Other

**FIGURE 3:**
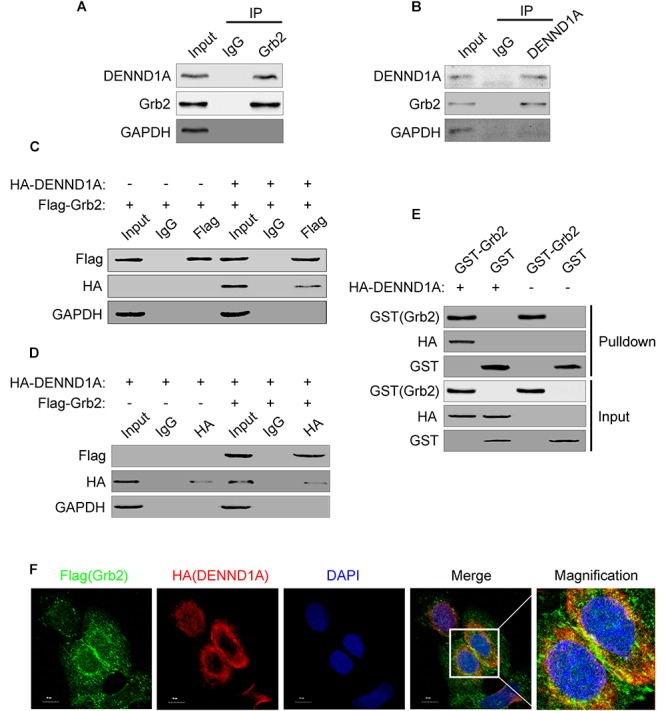
DENND1A and Grb2 combine with each other. **(A)** BGC-823 cells lysates were immunoprecipitated with an anti-Grb2 antibody or **(B)** anti-DENND1A antibody, and then both unprocessed lysates (Input) and immunoprecipitates were analyzed by western blot with indicated antibodies. **(C)** HEK-293T cells transiently transfected with Flag-Grb2 and HA-DENND1A were immunoprecipitated with an anti-HA antibody or anti- Flag antibody, and then both unprocessed lysates (Input) and immunoprecipitates were analyzed by western blot with indicated antibodies. **(D)** GST-Grb2 was incubated *in vitro* with lysates of HEK-293T cells transfected with or without HA-DENND1A, and coprecipitation of DENND1A with GST-Grb2, bound to glutathione-beads, was analyzed by western blot. GST, GST alone used as a control. **(E)** BGC-823 cells expressing Flag-Grb2 and HA-DENND1A were cultured on coverslips and fixed and double-stained with anti-HA and anti-Flag antibody. **(F)** Original colors were Flag-Grb2, green; HA-DENND1A, red; and nuclei, blue. Merged pictures represent the composite of all channels, with yellow regions indicative of colocalization. Scale bar, 10 μm. All experiments were repeated at least three times.

In view of the above results, we further studied the mechanism of EGF stimulation on DENND1A. DENND1A contains three DENN domains, clathrin-binding motif (CB), proline-rich domain (PRD) and AP-2 interaction motif (AP-2IM). According to reports in the literature, PRD and SH3 domains can bind to each other. We searched https://string-db.org and found that the downstream EGFR adaptor protein, Grb2, contains two SH3 domains at its N-terminus and C-terminus and a SH2 domain. And, studies have shown that Grb2 can bind to the PRD of SOS (Ras-GEF) through the SH3 domain, resulting in activation of SOS. Therefore, we speculate that DENND1A may be released from “self-inhibition” by interacting with Grb2 in a similar manner. We demonstrated that there is an interaction between endogenous DENND1A and Grb2 in BGC-823 cells by immunoprecipitation (Figures 3A,B). We also repeated this experiment on the SGC-7901 cell line and the results were consistent (Supplementary Figures S1A,B). In addition, both DENND1A and Grb2 could be combined with each other to form a complex by exogenous transfection and GST-pulldown (Figures 3C–E). Further studies confirmed that there was a significant co-localization of DENND1A and Grb2 in BGC-823 cells (Figure 3F).

### DENND1A Binds to the SH3 Domain of Grb2 Through PRD

**FIGURE 4:**
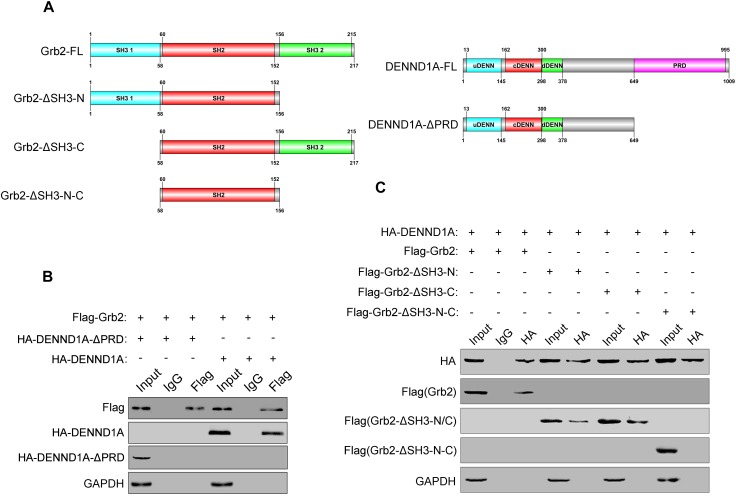
DENND1A binds to the SH3 domain of Grb2 through PRD. **(A)** Schematic diagram of different Flag-tagged Grb2 fragments and HA-tagged DENND1A fragments. **(B)** Coprecipitation of HA-tagged DENND1A fragments or **(C)** Flag-tagged Grb2 fragments with Flag-Grb2 or HA-DENND1A were analyzed by immunoblotting. Unprocessed lysates of HEK-293T cells transfected with Flag-Grb2 or HA-DENND1A was used as input. All experiments were repeated at least three times.

After demonstrating the interaction between DENND1A and Grb2, we further studied the binding sites of DENND1A and Grb2. We based on the known division of the DENND1A and Grb2 clones into a number of fragment fusion HA and Flag tags (Figure 4A), and transfected them in BGC-823 cells. We found that the DENND1A-FL fragment can bind to Grb2, whereas DENND1A-ΔPRD cannot. This indicated that DENND1A interacts with Grb2 through its PRD (Figure 4B). We also found that Grb2-FL, Grb2-ΔSH3-N, and Grb2-ΔSH3-C all bind to DENND1A, while the Grb2-ΔSH3-N-C fragment cannot, suggesting that both N- and C-terminal SH3 regions of Grb2 can bind to DENND1A (Figure 4C). Thus, DENND1A binds to the N-terminal and C-terminal SH3 domains of Grb2 through its PRD.

### EGF Stimulation Promotes EGFR Recruitment of Grb2-DENND1A Complex

**FIGURE 5:**
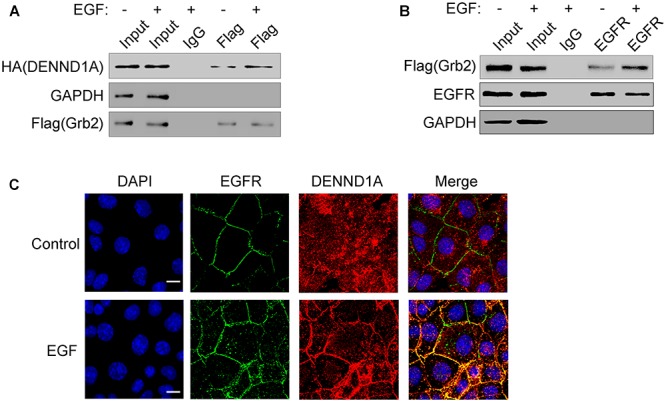
EGF stimulation promotes EGFR recruitment of Grb2-DENND1A complex. **(A)** BGC-823 cells transfected with Flag-Grb2 and HA-DENND1A were treated with or without EGF. Cells were lysed and analyzed for coprecipitation of DENND1A and Grb2 by western blot. Cells transfected with Flag-Grb2 and HA-DENND1A were used as input. **(B)** BGC-823 cells transfected with Flag-Grb2 were treated with or without EGF. Cells were lysed and analyzed for coprecipitation of Grb2 and EGFR by western blot. Cells transfected with Flag-Grb2 were used as input. **(C)** BGC-823 cells were treated with or without EGF and fixed and double-stained with anti-DENND1A and anti-EGFR antibody. Original colors were DENND1A or EGFR, red and nuclei, blue. Merged pictures represent the composite of both channels. Scale bar, 10 μm. All the experiments were repeated at least three times.

Studies have shown that EGF stimulation can cause EGFR phosphorylation. Phosphorylated EGFR can bind to the SH2 region of Grb2, thereby recruiting Grb2 to EGFR. We have demonstrated that Grb2 and DENND1A interact with each other in the above, so we further investigated the effect of EGFR signaling on DENND1A-Grb2. We tested the interaction of Grb2 with DENND1A and EGFR upon EGF stimulation. The results showed that compared with the control group, the EGF-stimulated group significantly enhanced the interaction between Grb2 and DENND1A or Grb2 and EGFR in BGC-823 cells (Figures 5A,B), indicating the interaction of Grb2 with DENND1A and EGFR. And, the changes of interaction depend on the EGF stimulation. The results of immunofluorescence showed that the DENND1A in the control group mainly distributed in the cytoplasm, while the EGFR mainly distributed in the cell membrane. DENND1A is mainly distributed in the vicinity of the cell membrane, and EGFR is also partially activated and endocytosed upon EGF stimulation for 5 min (Figure 5C). The results suggest that EGFR can recruit Grb2-DENND1A complex upon EGF stimulation.

### The Binding of Grb2 and DENND1A Affects the Activity of Rab35 and the Migration of BGC823 Cells

**FIGURE 6:**
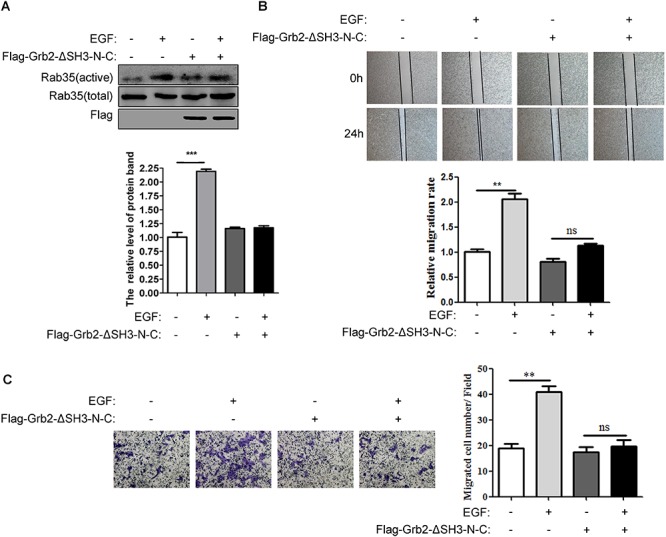
The binding of Grb2 and DENND1A affects the activity of Rab35 and the migration of BGC823 cells. **(A)** BGC-823 cells transfected with or without Flag-Grb2-ΔSH3-N-C were treated with or without EGF. The expression of endogenous activated and total Rab35 was analyzed by western blot. **(B)** And, the relative migration rate was calculated by normalizing the values obtained for the group of BGC-823 cells treated without Flag-Grb2-ΔSH3-N-C and EGF at 24 h as 100%. **(C)** Transwell assay was performed on BGC-823 cells transfected with or without Flag-Grb2-ΔSH3-N-C to evaluate the ability of migration and invasion. All experiments were repeated at least three times (^∗∗^*P* < 0.01; ^∗∗∗^*P* < 0.001).

Further, we verified whether the binding of Grb2 and DENND1A affects the change of Rab35 activity. The results showed that compared with the control group, EGF stimulation was performed after transfection with Flag-Grb2-ΔSH3-N-C, and the activity of Rab35 was not significantly enhanced (Figure 6A). In addition, wound healing and transwell assay showed that compared with the control group, the migration and invasion of BGC-823 cells transfected with Flag-Grb2-ΔSH3-NC upon EGF stimulation were inhibited (Figures 6B,C). We also repeated this experiment on the SGC-7901 cell line and the results were consistent (Supplementary Figures S1C,D). These results indicate that the interaction between Grb2 and DENND1A plays an important role in Rab35 activity and cell migration and invasion in gastric cancer cells.

### The Expression of DENND1A Affects the Prognosis of Patients With Gastric Cancer

**FIGURE 7:**
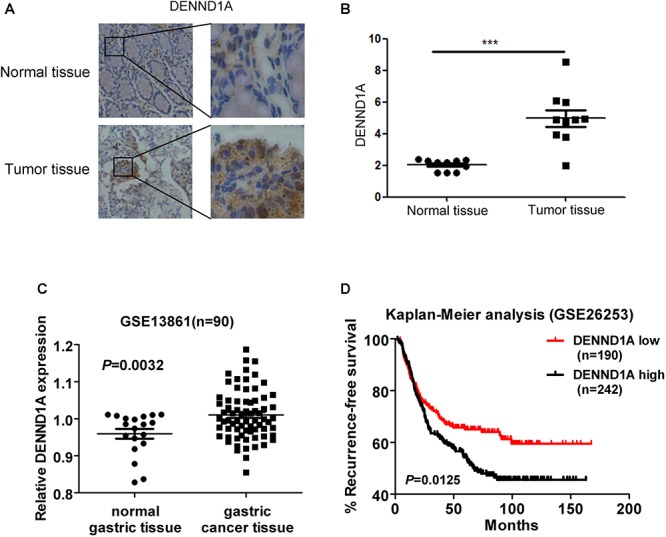
The expression of DENND1A affects the prognosis of patients with gastric cancer. **(A,B)** The expression of DENND1A was analyzed by immunohistochemistry in normal gastric tissue and gastric cancer tissue (*n* = 20). **(C)** The expression of DENND1A was analyzed by GEO database in normal gastric tissue and gastric cancer tissue (*n* = 90). **(D)** Kaplan-Meier survival plots demonstrating the poor prognostic effect of DENND1A gene high expression correlated with a short RFS in gastric cancer patients (*n* = 432). All data are log transformed and median centered (^∗∗∗^*P* < 0.001).

Finally, we verified that DENND1A expression was significantly higher in gastric cancer tissues compared to normal gastric cancer tissues by tissue microarray staining at the tissue level (Figures 7A,B). In addition, we analyzed the expression of DENND1A in gastric cancer tissues of the GEO database and found that the expression of DENND1A in gastric cancer tissues was significantly higher than that in normal gastric tissues (GES13861, *n* = 90, *P* = 0.0032) (Figure 7C). Further analysis based on database data showed that the higher expression of DENND1A, the shorter progression-free survival of gastric cancer patients, which indicated that the prognosis of gastric cancer patients with high DENND1A expression is worse (GES26253, *n* = 190, 242, *P* = 0.0125) (Figure 7D). This shows that the expression of DENND1A has a certain guiding significance in predicting the prognosis of patients with gastric cancer. At the same time, EGF-DENND1A-Rab35 signaling pathway with DENND1A as a regulatory center may also become a new target for the treatment of gastric cancer.

## Discussion

Gastric cancer is one of the most common malignancies in the world. Its morbidity and mortality have remained high on a global scale. According to statistics, deaths caused by gastric cancer are also increasing year by year. The high mortality rate of gastric cancer is mainly caused by its metastasis in the late stage. Therefore, it is important to study the mechanism of gastric cancer metastasis for the prevention and treatment of gastric cancer (Ferlay et al., 2015). The metastasis of tumor cells is a complex process that integrates multiple changes in the signal pathways of cells, including PI3K-AKT, Ras-MAPK, STAT, P38 and Juk-SAPK, which trigger tumor cells migration and invasion. In many of the above signaling pathways that regulate the migration of tumor cells, PI3K-AKT, Ras-MAPK, and STAT signaling pathways can all accept the stimulation of EGFR (Hong et al., 2014). EGFR contains an extracellular domain, a transmembrane domain, and an intracellular tyrosine kinase activity domain. EGF binds to the extracellular domain of EGFR and activates it, resulting in phosphorylation of the intracellular tyrosine kinase, which causes downstream a series of signaling pathways which involved in various stages of tumor progression, such as cell proliferation, angiogenesis, invasion, migration, metastasis, and apoptosis (Hong et al., 2014; Jacome et al., 2014; Yuan et al., 2017).

Our previous studies have shown that in non-small cell lung cancer and cervical cancer cell lines, EGF stimulation can promote intracellular Rab35 activity, thereby promoting tumor cell migration or proliferation (Duan et al., 2016; Zheng et al., 2017). Consistent with these research results, our study in gastric cancer also showed that EGF stimulation can also promote the activation of Rab35, and thus promote BGC823 cells migration. As an important small G protein in the cell signaling pathway, Rab35 is one of the members of the Ras family and its activation is regulated by its GEFs. Studies have shown that FLCN as an GEF for Rab35 in Hela cells has a significant regulatory role (Zheng et al., 2017). In addition, there are no more studies on the upstream regulatory mechanisms of Rab35 in tumors. In view of this, we have conducted further studies in gastric cancer. We found that DENND1A is highly expressed in gastric cancer cells by screening the expression of GEFs in gastric cancer cell lines. At the tissue level, we also obtained the same conclusions through immunostaining of tissue microarrays and analysis of gastric cancer RNA sequencing data in the GEO database. Compared with normal gastric tissue, DENND1A showed a significant high expression in gastric cancer tissues. Moreover, the high expression of DENND1A significantly shortened the progression-free survival of patients with gastric cancer. So its expression has important guiding significance for the prognosis of patients with gastric cancer. These all suggest that Rab35 activated by DENND1A may play an important role in the development of gastric cancer.

The role of DENND1A is rarely reported in the development of tumors. Studies have shown that DENND1A is highly expressed in patients with polycystic ovary syndrome (PCOS) and plays a catalytic role in its development (Eriksen et al., 2013; McAllister et al., 2014). In addition, no studies have revealed its role in tumors. However, the same family protein FLCN has been studied in breast cancer, kidney cancer and cervical cancer (Zheng et al., 2017; Hou et al., 2018; Sattler et al., 2018). In view of this, our studies in gastric cancer have shown that DENND1A exerts GEF action during the activation of Rab35 stimulated by EGF, and through its interaction with Grb2, it can regionally recruit activated Rab35 and then promote the migration of tumor cells. Grb2 as an important adaptor protein is the downstream of EGFR and contains both SH3 and SH2 domains. In addition to the DENN domain necessary for activating Rab35, DENND1A also contains a PRD domain that can interact with the SH3 domain. Our study confirms that EGF-activated EGFR interacts with DENND1A through its downstream Grb2 interaction, which is achieved through the interaction of the SH3-PRD domains, and thus recruited DENND1A and activated Rab35 molecules by this. According to this, the tumor cells migration is promoted, and local stimulation and activation of signaling molecules also provide a certain direction for the cells migration.

Previous studies have shown that Rab35 can regulate a variety of intracellular signaling pathways and a variety of cytological phenomena. Studies have shown that activation of Rab35 during multiple cellular events can promote its effector molecules, such as OCRL, Fascin, and ACAP2, recruit to a specific area of the cell, and then function (Zhang et al., 2009; Dambournet et al., 2011; Kobayashi and Fukuda, 2012; Allaire et al., 2013). Rab35 can act as an upstream of PDK1 and mTORC2 during tumorigenesis and development, and it can act in conjunction with PI3K (Wheeler et al., 2015). Therefore, we did not make too many statements about the downstream regulatory events of Rab35. In this study we demonstrated for the first time that EGF stimulates EGFR and activates Rab35 via DENND1A in gastric cancer cells. Therefore, our study of the upstream regulatory mechanism of Rab35 is a good complement to the role of Rab35 in tumor development. It is the upstream activation and recruitment that determines the diversity of Rab35 in regulating tumor progression. Our study also demonstrated this in the migration and invasion of gastric cancer cells.

In summary, we confirmed that EGF-induced activation of Rab35 in gastric cancer cells is regulated by DENND1A in this study. In the process of gastric cancer cell migration, EGF activates EGFR, which through the combination of Grb2 and DENND1A plays a role in target molecule activation and targeted recruitment. The binding of Grb2 and DENND1A were also confirmed through SH3 and PRD domains. Upon EGF stimulation, the binding increases and promotes the activation and recruitment of Rab35. In the process, Rab35 undertakes the stimulation and further promotes the migration and invasion of gastric cancer cells through its various downstream signaling molecules. Ultimately, the expression level of DENND1A predicts the survival status of gastric cancer patients and may become one of the important targets for the treatment of gastric cancer.

## Author Contributions

BY and BD designed the study. BY, WD, YW, and YC performed the experiments. BY and SS performed the statistical analysis. YZ, JD, and BD drafted the manuscript. JC, YZ, and LG supervised the experimental work. All authors read and approved the final manuscript.

## Conflict of Interest Statement

The authors declare that the research was conducted in the absence of any commercial or financial relationships that could be construed as a potential conflict of interest.

## Supplementary Material

FIGURE S1 Partially important experiments were repeated in the SGC-7901 cell line. **(A)** SGC-7901 cells lysates were immunoprecipitated with an anti-Grb2 antibody or **(B)** anti-DENND1A antibody, and then both unprocessed lysates (Input) and immunoprecipitates were analyzed by western blot with indicated antibodies. **(C)** SGC-7901 cells transfected with or without Flag-Grb2-ΔSH3-N-C were treated with or without EGF. The expression of endogenous activated and total Rab35 was analyzed by western blot. **(D)** And, the relative migration rate was calculated by normalizing the values obtained for the group of SGC-7901 cells treated without Flag-Grb2-ΔSH3-N-C and EGF at 24 h as 100% (^∗∗^*P* < 0.01).

TABLE S1 Detailed tissue case information for 20 tissue chip samples.
