# Supplementary figures and images for: EGF Stimulates Rab35 Activation and Gastric Cancer Cell Migration by Regulating DENND1A-Grb2 Complex Formation
Source: Front Pharmacol. 2018 Nov 22;9:1343. doi: 10.3389/fphar.2018.01343 (PMC6261971; doi:10.3389/fphar.2018.01343)

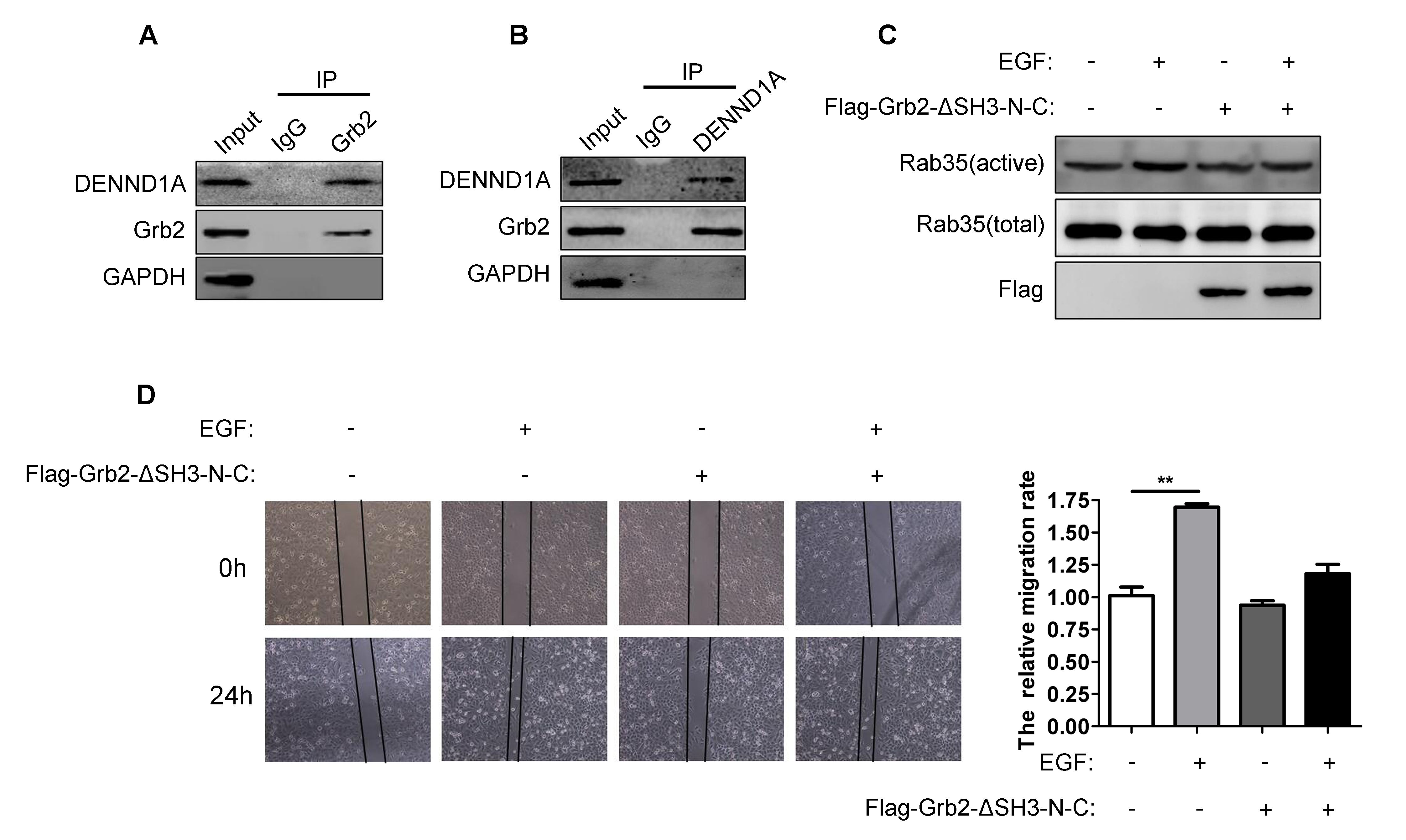

Supplement: FIGURE S1 — Partially important experiments were repeated in the SGC-7901 cell line. (A) SGC-7901 cells lysates were immunoprecipitated with an anti-Grb2 antibody or (B) anti-DENND1A antibody, and then both unprocessed lysates (Input) and immunoprecipitates were analyzed by western blot with indicated antibodies. (C) SGC-7901 cells transfected with or without Flag-Grb2-ΔSH3-N-C were treated with or without EGF. The expression of endogenous activated and total Rab35 was analyzed by western blot. (D) And, the relative migration rate was calculated by normalizing the values obtained for the group of SGC-7901 cells treated without Flag-Grb2-ΔSH3-N-C and EGF at 24 h as 100% (∗∗P < 0.01). [file Image_1.TIF]
